# Cured of Primary Bone Cancer, But at What Cost: A Qualitative Study of Functional Impairment and Lost Opportunities

**DOI:** 10.1155/2015/484196

**Published:** 2015-04-09

**Authors:** Lena Fauske, Oyvind S. Bruland, Ellen Karine Grov, Hilde Bondevik

**Affiliations:** ^1^Department of Oncology, Oslo University Hospital, Norwegian Radium Hospital, P.O. Box 5960, Nydalen, 0424 Oslo, Norway; ^2^Institute of Clinical Medicine, University of Oslo, P.O. Box 1078, Blindern, 0316 Oslo, Norway; ^3^Faculty of Health Sciences, Department of Nursing, Oslo and Akershus University College of Applied Sciences, St. Olavs plass, P.O. Box 4, 0130 Oslo, Norway; ^4^Institute of Health and Society, Department of Health Sciences, University of Oslo, P.O. Box 1089, Blindern, 0317 Oslo, Norway

## Abstract

*Purpose*. Our study aims to explore how former cancer patients experience physical and psychosocial late effects 3–7 years after they underwent treatment for primary bone sarcoma in the hip/pelvic region. A qualitative, phenomenological, and hermeneutic design was applied. *Methods*. Sarcoma survivors (*n* = 10) previously treated at Oslo University Hospital, Norwegian Radium Hospital were selected to participate. In-depth and semistructured interviews were conducted. The interviews were analysed using inductive thematic analysis. *Results*. The participants reported that the late effects had three core spheres of impact: “their current daily life,” “their future opportunities,” and “their identity.” They expressed negative changes in activity, increased dependence on others, and exclusion from participation in different areas. Their daily life, work, sports activities, and social life were all affected. Several of their experiences are similar to those described by people with functional impairment or disability. *Conclusion*. Patients cured of bone cancer in the hip/pelvic region pay a significant price in terms of functional impairment, practical challenges, exclusion from important aspects of life, and loss of previous identity. It is important to appreciate this in order to help bone cancer survivors who struggle to reorient their life and build a secure new identity.

## 1. Introduction 

Primary bone sarcomas are rare and represent less than 0.2% of all new cancers [1], with osteosarcoma, chondrosarcomas, and Ewing's sarcoma being the most common entities [2]. Whereas osteosarcoma and Ewing's sarcoma occur mainly in children and adolescents, chondrosarcoma is most commonly diagnosed in adults [3–5]. Cure of bone sarcomas in the hip/pelvic region may require extensive surgery, often in combination with chemotherapy and radiotherapy [2].

In general, former cancer patients frequently exhibit a poorer health status than those who have not had cancer. Studies show that the late effects of therapy may cause functional impairment, pain, and psychosocial challenges [6–12]. Fatigue and reduced cognitive function [13–15], changes in sexuality and reproduction [10, 16–18], changes in body image [19–23], and challenges in terms of educational and vocational life [24–27] as well as a negative impact on leisure activities and social life [10, 11] are all consequences that can affect former cancer patients' daily lives. These late effects can last for months or years after the patients are seemingly cured of cancer or may only occur many years later [7, 28]. This implies that former bone sarcoma patients may experience considerable health challenges and functional impairment as long lasting consequences of their treatment [29–31].

To our knowledge, no studies have addressed how the practical and psychosocial lives of former bone cancer patients who have undergone extensive surgery in the hip/pelvic region are perceived by the patients themselves several years after treatment. There are some functional [32, 33] and a few qualitative research publications concerning bone cancer in the lower limb [34, 35] as well as one on sacral cancers [36]. These studies show that functional impairment and fatigue affect activities in both daily living and social life, including an active outdoor life [35, 36] as well as the ability to have a vocational life [34, 37, 38]. Since many of the patients affected by bone sarcoma are young, any long-term effects of treatment can be expected to significantly impact their future opportunities. This has also been documented among children and adolescents cured of other cancers [10, 27, 39]. Drew states that children and adolescents, in particular, go through an extensive reorientation to life as part of their rehabilitation process [39]. Recovering from cancer does not necessarily mean that the patients are done with cancer but rather that they enter a new phase of life with different conditions than before. Drew's study illustrates how the original disease as well as the associated treatment influences each cancer survivor's long-term health, life-course experiences, and social interactions [39]. Parsons et al. report that their subjects, all of whom had been affected by bone cancer in the lower extremities, engaged in three types of “work” after getting their diagnosis: “illness work” when going through treatment and struggling with consequences, “identity work,” and “vocational work.” Importantly, participants in their study described an active process of “identity work,” work that is characterised by “becoming other” through self-reflection and effort [34]. Despite the late effects and challenges experienced by former cancer patients, several studies also report positive growth and beneficial outcomes [40–43].

Cancer can be understood as a serious incident in life and as a biographical disruption [44–46] as well as a loss of self [47]. First of all, the cancer patient is a person for whom illness has broken into their daily life and changed their life experience. The old story of their life is broken as is their identity [44, 45, 48]. When affected by a serious disease and its consequences, people often have to reorient their life and construct a new story with their affected body as a new starting point. This helps to make sense, both in terms of understanding themselves and the world of illness and recognising new limitations in their lives [44, 49].

Our study is based on interpersonal encounters. The aim is to understand the experience of illness through qualitative methodology. We interviewed the vast majority of eligible former sarcoma patients who met the inclusion criteria. They were all treated at Oslo University Hospital, Norwegian Radium Hospital (OUS NRH). Their stories were analysed, highlighting how late effects have affected their current daily life as well as influencing their future possibilities and their identity.

## 2. Methods 

To explore how former sarcoma patients experience life after cancer, we have applied phenomenological experience-based and hermeneutics interpretation-based perspectives on disease. In phenomenological research, the aim is to investigate individual human experience (phenomena) as manifested in daily life and in specific situations [46, 50–52]. Hermeneutics is about how to achieve understanding and how phenomena have to be interpreted in order to be understood. Here, comprehension develops through the entire process and is based on both participant's and researcher's preunderstanding and the historical and cultural context [53, 54]. This will impact on the questions raised, the interview process, its transcription, and the subsequent analysis [51, 53, 55]. Svenaeus, based on the philosophy of Heidegger [52], has developed a phenomenological approach to disease [45, 46]. He claims that serious illness forces people to reorient to life. Patients' ailments are multidimensional phenomena and, at the same time, meaningful on several levels [46]. We attempt to connect the biological concept of disease with patients' experiences as well as both the psychosocial and sociocultural aspects of illness.

### 2.1. Participants and Recruitment

Potential respondents were identified in the prospective clinical sarcoma database (MedInsight) at OUS NRH, which treats approximately 80% of patients with bone sarcomas in Norway [56]. Of the 12 eligible patients contacted, 10 agreed to participate.

Seven men and three women, aged between 18 and 60 years (see Table 1), who had been treated for bone sarcoma in the hip/pelvic region participated in this study. They represent a range of ages and have diverse backgrounds. They all write and speak Norwegian. All were treated with surgery: three had hip transposition, one saddle-prosthesis, one fibula-graft reconstruction, four only tumour resection without reconstruction, and one a proximal femur mutars-prosthesis. Seven participants received chemotherapy, while two had additional radiotherapy. One participant subsequently had an amputation with hemipelvectomy due to chronic infection. All participants were diagnosed between 2004 and 2009 (see Table 1) and were followed up at the oncological or orthopaedic surgical outpatient clinic at OUS NRH. None experienced a relapse of the disease for at least three and up to a maximum of seven years following primary diagnosis. Initial contact with the participants was made by the treating physician at OUS NRH. The first author then gave further details regarding the project before the participants provided informed consent. The interviews were conducted face-to-face by the first author in connection with a routine clinical follow-up appointment that took place at OUS NRH.

### 2.2. Procedure

This research is anchored in the fundamentals of the Helsinki declarations. Permission to conduct the interviews as well as to collect and store sensitive data was obtained both by our Institutional Review Board and the Regional Committee for Medical Research Ethics, REK South East. All information was stored confidentially. The analyses were carried out from anonymised transcripts.

The interviews had an average duration of 66 minutes (ranging from 31 minutes to 102 minutes) and were audiotaped and then transcribed verbatim by the first author (8) and a medical secretary at OUS NRH (2). Field notes were written following each interview in order to document observations made by the interviewer. The interview guide consisted of the following topics: how the participants experienced their cancer diagnosis and treatment; functional, practical, psychosocial, emotional, and vocational consequences of the disease and treatment; and whether the cancer experience has changed them as a person. In this paper, we focus on how functional impairment, pain, and fatigue have influenced their lives. The interview guide was designed to allow the participants to tell their whole cancer story chronologically. The interview guide was, however, only loosely followed, so that the participants were able to highly influence the depth of the interview. Through this approach, structure and meaning are produced jointly by the participant and researcher [57]. As such, certain interpretations emerged during the interview from both sides. This enabled the confirmation or rejection of the interviewer's perceptions of what the participants expressed [51].

### 2.3. Data Analysis

The participants' accounts were analysed by hand by the first and last author using thematic analyses. The analysis took place in stages and followed an inductive strategy [58] within a contextualised framework [51]. First, the transcribed interviews were read through to gain an overall impression and to identify preliminary themes. Second, the entire data set was coded in detail and then organised into themes, and concepts were developed. The themes were reflected on in accordance with the study's objectives and were compared against the available literature and theory that highlights the interaction between cancer and the patient's life experiences.

## 3. Results

This study shows that both somatic consequences and psychosocial experiences present several challenges to former sarcoma patients. These effects are manifested in practical terms, in work and leisure times, and in their emotional and social life (see Table 2). The price paid for a cure leads to several changes, losses, and challenges; life is not the same as before. These survivors may be excluded from several contexts that were important in their lives before cancer. We have identified three main findings in this study: the impracticalities of daily life due to functional impairment, lost opportunities, and an identity change.

### 3.1. The Impracticalities of Daily Life

Despite previously being healthy and active, most participants now suffer different functional impairments that limit their daily lives. Several described how cancer has affected and changed their lives in fundamental ways and how they now require different kinds of support.

Six of the ten participants require technical aids to ambulate due to problems related to poor balance, decreased strength, or lack of mobility after the removal of their bone sarcoma, for one of the participants, an amputation. These six are dependent on crutches or a cane and two of them have to use a wheelchair. Stairs therefore pose a formidable challenge for many of them. P3 (participant #3), for example, has one leg 7 cm shorter than the other and with muscles removed. Hence, he has less strength in that leg and poor balance. As he expressed:* I use crutches to avoid pain and to be able to walk faster. I walk very slowly and with difficulty without crutches. […] Of course, I cannot climb stairs without crutches.* Some participants also stated that poor balance and functional impairment prevent them from taking public transportation. They are now dependent on taxis or on having a car of their own. Most of them need a car with automatic transmission and a few require specially adapted vehicles. In Norway, snow and ice present severe challenges to getting around during several months of the year for those with poor balance and diminished strength. Most of the participants are afraid of falling because their prostheses can be damaged. Carrying bags or heavy things is difficult. P5 recounted how this is experienced:* It is obvious that I am more helpless; I need more help* –* definitely.* She also said that it has become challenging to go into the city alone. She prefers to have an arm to lean on in order to feel safe. The fear of being knocked over is real and it is difficult to carry anything. She also emphasises that the extent of her disability is not obvious to someone looking at her:* My functional impairment is, in fact, more than what people can see from watching me walk.* That makes it difficult for others to realise the challenges she faces and what they ought to therefore take into consideration.

When certain movements are limited or no longer possible, housework also presents formidable challenges. It may be impossible to do chores that involve bending over or accessing hard-to-reach places. Standing on a chair is difficult. Household chores may be not only painful and difficult but also time consuming. P10 stated:* I cannot get anything done (on crutches). […] I cannot carry anything; I cannot take anything with me. I have to put everything in a rucksack. For example, making dinner on crutches and trying to carry hot pans is absolutely hopeless.* For him, the alternative is to perform most of the work he does at home from a wheelchair, which presents a whole new set of limitations. For many, this means that some housework is cumbersome and very strenuous to manage on their own. As a consequence, some work is postponed or not even done. Many of the participants have to ask others for help to get around in their daily lives.

Radical surgery on a bone sarcoma in the hip/pelvic region may result in stomata. P10 has both a colostomy and a urostomy. He pointed out that there are no merely practical challenges to having these, but also how stomata drain his energy when he is travelling or stays outside his home:* The urostomy is more difficult because there are often leaks and it is difficult to avoid them. […] Then I have to find a lavatory and go in and change. I always carry a bag with extra equipment and clothes. […] I never go out the door without it. That translates into a lot of bother, leading to delays and it wears me out as it takes a lot of energy*. For some of the participants, ordinary tasks like going to the bathroom or taking a shower can no longer be taken for granted. According to 4 out of the 10 participants, poor balance calls for armrests on the toilet or a special shower stool. As P9 explained:* The shower chair also has to be brought along if I am staying anywhere else than home.*

There did not seem to be any discrepancy between adolescents and adults in terms of the daily practical challenges faced.

### 3.2. Lost Opportunities and an Altered Future

One common denominator for many of the participants is that they were physically active before being diagnosed with cancer. Active lives, sports, and outdoors activities were important aspects of their lives. However, following treatment, it is no longer possible for several of them to engage in activities such as bicycling, jogging, skiing, playing football, tennis, outdoor life, or hunting. Accordingly, many feel cut off from an important part of their lives and from activities that previously helped fill their lives with content and meaning. Due to impairment, several participants have not had the possibility of getting involved in a new hobby involving physical activity. P2 was a semiprofessional athlete before getting sick:* I have no new hobby to replace the sport that was my passion.* At another place in the interview, he acknowledged:* One sits around and lives on the memories, even at the age of 30*. The study clearly found that those who had not had a physically demanding hobby before being diagnosed with cancer did not feel the same sense of being cut off from this part of their lives.

The participants' social lives are also affected when they are unable to participate in activities in the same way as before. Many of them state that they are left standing on the side-line or are left out. P4 was in his teens when he was diagnosed with cancer. He felt it was especially difficult to not be active along with other teenagers:* I cannot go skiing or play football. […] I do feel it is sad.* P3 stated that he could have spent more time with others and participated in several activities if he had not been dependent on crutches:* Many times, I have felt that if I were without crutches, I could have walked normally and even run. Then I could have taken part in more activities and possibly even had more friends*. Other participants also recounted how reduced functionality, fatigue, and pain have impacted on their social lives. P6 stated explicitly that long-term consequences of her treatment have made her less social:* I spend less time with friends than I used to do. I feel sad about that. I feel it is the pain that is my biggest problem.*

The experience of loss and the feeling of uncertainty about the future are also linked to vocational life. For the majority of those included in this study, employment or studies account for a significant part of their lives. In our study, only two participants have had to give up working completely as a result of their cancer. When not employed, it is easy to become an outsider in many important aspects of life. P6 had to quit working in the restaurant industry as the job required physical mobility. Losing her job was the greatest change in her life after having cancer. She would still prefer to work, even though she cannot work full-time. Being outside the workforce makes her feel isolated and has posed challenges to her mental health:* I need something like work to go to, if not, anxiety will reappear.* She dreams of working for a few hours a week, but unpredictable health with pain and fatigue makes it difficult to get an employer to hire her.

P7 is a student at the moment and worries about her future job opportunities. She is on the threshold of adulthood, and she is training to work in tourism and dreams of a career in which she can use her education. Today she is dependent on crutches. As the situation is now, she does not feel very attractive for the labour market. As she reflected:* No one wants to hire anyone on crutches.* She hopes that having a leg extension will make her more mobile, but she also knows that the rehabilitation process can be both lengthy and difficult before she reaches her goal, if it is at all possible.

Both adolescents and adults expressed concerns about lost opportunities, although an altered future would arguably have the greatest impact on the youngest survivors.

### 3.3. I Am No Longer the Person I Once Was

The stories related in the interviews indicate that the participants' individual identities have been affected. The participants have gone from being healthy to being labelled by their disease, not as sick, but as disabled, not completely healthy or capable of functioning. As P3 articulated:* I am, of course, defined as being disabled.* In this study, the male participants' experience of themselves and their understanding of their own identities are expressed as the juxtaposition between what they say about sports, outdoor activities, work, and social relationships on the one hand and the young men's understanding of their own masculinity on the other. However, none of the female participants expressed that their gender identity was influenced as a consequence of being functionally impaired.

P2 was a semiprofessional athlete before being diagnosed with cancer. Today, he is not sufficiently functional to engage in sports, although he spends a lot of time working out at the fitness centre. This is essential for regaining functionality and strength after surgery. He does not try to hide that it has been mentally demanding to go from being an elite athlete to being physically impaired:* My identity was closely linked to being an athlete. […] I have had to work really hard to carve out a new identity. […] At least, I now feel as though I'm making a start. […] And I understand that people like me even if I'm not playing sports.*

For generations of Norwegians, identity has been associated with skiing and an active outdoor life. No one can tell from looking at P4 that he can no longer go skiing. He has tried after recovering but has no control and so just falls. He is afraid that others will consider him lazy or not very sporty. This negative change in identity seems to bother him:* I'm embarrassed because I could do this before, but of course I cannot prove that now. This has something to do with identity* –* I feel a little less cool, less sporty and somewhat less of a man. […] It is a kind of stupid, but it makes me focus on school to a much greater extent*. P4 stated that he has found a way to cope; he now does exceptionally well with his studies.

In P9's case, we see how important it is to get the opportunity to reorient to life after extensive cancer treatment. He eventually had a leg amputated involving a hemipelvectomy. Prior to that, he was sick for several years due to postsurgical infections. This prevented him from entering a new life as a cured and healthy person. He is today totally dependent on other people, especially his wife. Before the cancer, he spent a lot of time outdoors hunting and fishing. His life and identity were linked to being an active outdoorsman:* I am no longer an outdoorsman. […] I have nothing, and I do nothing.* We recognise here that a prerequisite for imbuing new meaning in life after cancer is that one is sufficiently healthy to embark on this process. This is a process that calls for energy and spirit. In the case of P9, the adverse consequences of the cancer treatment were so extensive that he has not started on the process of reinventing himself. For him, life is still a struggle.

Before cancer struck, P10 was a young man who devoted all his spare time to organisational work and not sports. Despite now being nearly always confined to a wheelchair, he is still active in local organisations. With crutches, he can move over short distances, but this results in pain and exhaustion. Although a wheelchair is more practical, people's prejudices make him choose crutches more often than he would like. For him, it is important to be recognised as the positive resource he actually is and not as someone failing to contribute because he sits in a wheelchair. He dislikes how people give him an identity as a wheelchair user and not a person with strong resources and as an important contributor:* When joining meetings I use crutches because this give a completely different image […] many have a theory that you have brain damage if you sit in a wheelchair. […] They see the wheelchair and not the man.* He states that, if he had sufficient energy, he would have taken on the battle against people's prejudices.

The younger participants (*n* = 6) claim that experiences following their cancer diagnosis have also positively influenced them as human beings, despite all the physical challenges. P3 acknowledged that the cancer has given him an opportunity to reorient to life. Despite the fact that he sees himself as a person with disability, with all the challenges that are implied, the cancer has done more good for him than bad. He is young and feels he has become a totally different person, a more positive and grounded person:* Many aspects of my life have changed as a result of having cancer. I used to be lazy. I was more serious. […] I was not very happy, took no joy in things, actually. Guess I felt that I was a bit boring. And I did not do much. I wasn't interested in much either. Not even curious. After having had cancer, I have become more positive, a little happier, and I have changed my opinion of myself. What I went through, and the fact that I was as strong as I was, gave me more zest for life. I am more appreciative of life. I sort of turned into a totally different person.* Four of the young participants were grateful for the positive experiences that the cancer had given them. Three of them went so far as to say their cancer has had more positive consequences than negative. As P10 stated:* Even though I sit in my wheelchair and have many bad days, I still believe that my cancer experience was ultimately beneficial for me.*

Several participants stated that staying in a job is important for their identity. P10 now holds 15% of an average full working week. However, to do that job, he actually has to be at work for at least twice that long:* Having a connection to work is important. It is not crucial for me to work full-time […] but rather to have an affiliation to my employer. […] Having a job makes a person feel like he or she is contributing to society. Then there is the question of what others think: Do you work or not? No, I am on disability benefits. I just cannot buy that at all.*

P5 received full disability benefits after a long career. She used to like her job and would have liked to continue with it, but fatigue, cognitive problems, reduced functions, and poor balance made it impossible:* My days are ups and downs, and I know that I tire quickly from things that never used to wear me out.* P5 also related that it was not possible for her to have a job and also to have enough energy for a social life. When she tried to work after having cancer, it took all of her energy. Today she has accepted that disability benefit is the best solution for her, although she is not comfortable with it. She wishes that she still had an identity as an employee.

The adolescents expressed more concerns than the adults about changes to their identity related to disability, sports, and masculinity. The exception to this was concerns about their work identity, which were expressed by several survivors regardless of age.

## 4. Discussion 

In this study, we have addressed how the late effects of extensive treatment of bone sarcoma in the hip/pelvic area, especially surgery, have impacted former cancer patients cured of their disease three to seven years earlier. Besides both physical and functional impairments, most of the participants also reported changes and losses as well as lost future opportunities. Life is radically changed, with an impact on their vocational work, social life, and participation in leisure activities as well as their identity. Our findings are in line with other qualitative bone cancer researches [34–37]. Unlike many other cancer patients, our group expressed consequences and challenges that are similar to those experienced following traffic or sports accidents. They face barriers and challenging social structures that provide exclusion from activities they would have had access to without their cancer history. Below, we will discuss some of the implications of being a person with disabilities following bone cancer treatment.

In this study, several participants expressed alterations in terms of the experience and perception of who they are. The change from being healthy and employable to being functionally impaired emerged as important, in terms of both personal identity as well as practicalities. Among our participants, we observed changes in activity, dependence on others, and exclusion from participation in different arenas, similar to the experiences described by people with a disability [59]. In the scientific literature, disability is an umbrella term. On one side, functional impairment is referred to as a property of the individual, based on an individual and disease-related understanding. In the social model, however, the focus is not on disability as an individual defect but more on the interplay between subjective function and ambient requirements, social injustice, and changes in the social and physical environments. Disability comprises both the individual's reduced function with limitations in activity on one hand and limitations in participation in different arenas and in different life situations on the other [60]. In a society grounded on being healthy, a person with a functional impairment or disability could be banned from participating in certain activities or situations [61], as experienced by many of our participants.

Research indicates that having a functional impairment after cancer may lead to exclusion from the labour market in general or certain jobs in particular [37, 38]. In our study, some participants found that reduced mobility, pain, fatigue, and cognitive impairment decreased their ability to be fully or partially employed. Employment is one of the most important factors for social integration and contributes to defining people's social status and personal identity [62]. Research indicates that being excluded from employment as a result of cancer can affect people's everyday structure, a structure that is built around work and that gives life richness and meaning. Furthermore, this could also result in a reduced social life, in addition to a loss of identity [24]. A change from being employable to being totally or partially incapacitated from work can be a challenge in a country like Norway, where most of the adult population is in work and being employed constitutes the central norm. Being an outsider can be difficult to reconcile. For young people, the late effects of cancer could have an impact on their study and work opportunities in the future [27]. As one of our participants also noted, one is not necessarily as attractive as an employee after having undergone extensive bone cancer treatment [34].

Physical activity is important for both people's well-being and good health. Research indicates that physical activity has a positive effect on former cancer patients' life and health [63, 64]. Several of the participants in this study state that they are less physically active now. This may involve more than just inactivity. For some, this will lead to less social activities than before the cancer struck, in line with other publications [35, 36].

In particular, those participants in our study who had immersive sports or outdoor interests before getting cancer experienced a loss that profoundly influenced several aspects of their lives. For previously active young men, being cut off from participating in sports activities can make them feel less masculine. According to Connell [65], the ability to participate in sport is seen as an important aspect of masculinity. It is not just about the loss of a hobby and social environment; it is also about the loss of identity that says something about who you are and who you want to be. Illness can affect man's status in masculine hierarchies and create doubt about his own masculinity [66].

A change in social status from being healthy to being a person with a disability is a change in identity and might be perceived as a loss and so cause social challenges. This is due to how you see yourself and how others treat you [59]. Being disabled makes you deviate from the normal, which may be stigmatising [67]. Stigma in general means that a person is thought of as being “other.” This can have many negative effects for those affected. They face prejudice and discrimination and are victims of stereotypes [68]. It is not uncommon to see people with disabilities as more dependent on others and as less intelligent [69]. One of our participants who uses a wheelchair faces such prejudices when he says that “they see the wheelchair and not the man.” This makes him use crutches in specific situations even though he can hardly walk.

Although a former bone cancer patient may face many negative changes and losses, it may also involve positive growth. The hurdles such patients have overcome and their work adapting to the new challenges may also have impacted positively on their life and identity. In the literature, such positive changes are termed as posttraumatic growth and characterised as a changed sense of oneself, a changed sense of relationship with others, and a changed philosophy of life [70]. Studies report positive growth among former cancer patients, especially those who received their diagnosis a few years earlier, and among those who are young [40, 41]. Among cancer patients, higher growth, both with increasing time from diagnosis and a younger age [40, 41, 71], has been found. Thornton's review of benefit findings in the cancer experience reports that a “substantial proportion” of previous cancer patients link positive changes in life perspective and relation to others and to self to their cancer and illness experiences [72]. In some studies of former adolescent cancer patients, the majority report positive growth [42], including a study of long-term survivors of extremity osteosarcoma [43]. The last questions in the interview guide gave them an opportunity to reflect on their current situation. Here the participants also expressed experiences of positive growth, after we had talked mostly about their challenges following cancer treatment. Only the adolescents and young adults talked about the positive changes cancer has had for them as individuals. To our surprise, the two young participants who considered themselves to have experienced pronounced growth were among those who suffered the worst late effects and physical challenges. Our study was not designed to address the cause of such a paradox, although it would certainly be a worthwhile focus for subsequent study.

As mentioned before, being cured of cancer is not always about getting back to the life they had before but it also requires that they reorient themselves to a new life with an altered body. To construct such a new identity, one must complete illness work [34]. This presupposes having the energy to establish a new understanding of themselves where the new body and the new conditions are integrated as part of that new identity. Accepting changes and reconciling them can be crucial to achieve resilience or growth. We note that one of the participants in our study in particular is still struggling after years of complications from treatment, a challenge also previously reported [73]. Our participant was never given the opportunity to build a new life with the new conditions and his new identity as a disabled person. The result is that he today says he has “nothing.” When all one's time and energy are devoted to cope with the complications of disease, it is difficult to shift focus and to reorient to life.

It is important to understand the challenges and struggles that this group of cancer survivors face. However, it is vital to not have a unilateral focus on negative late effects following treatment, as research shows that many cancer survivors, in addition to the aforementioned challenges, will find coping strategies that give them a good life despite the limitations they may have [74].

The small sample size may limit the generalizability of this study, although the majority of eligible participants did take part. However, in qualitative research, we are not looking for representative data but rather aim to illuminate the phenomena that the participants express. Nevertheless, when exploring cancer survivors' life experiences, we suggest using mixed methods to a greater extent. Qualitative and quantitative approaches together will gain a more complete picture of the consequences of cancer treatment. Another limitation is that we have only interviewed the participants once. Several interviews during a longer period from the time of diagnosis might have provided a more complete picture. In addition, prospective studies exploring how bone sarcoma survivors experience changes in sexuality and reproduction, as well as bodily deviations, are warranted.

## 5. Conclusions

Functional impairment that results in daily practical challenges, exclusion from important aspects of life, and loss of previous identity may be the price to pay for being cured of bone cancer in the hip/pelvic region. As osteosarcoma and Ewing's sarcoma particularly affect young people, a diverse range of life stages in which illness can affect and disrupt developmental milestones exists. It seems that such knowledge is important to help bone cancer survivors who struggle to reorient to life and build a new identity founded on their new situation in life. In order to advise the caregivers of those with sarcoma, the findings from this study suggest a systematic approach to long-term follow-up including provision of information, supervision and training regarding practical options, and social support. The assessment of each individual survivor's situation seems vital and so appropriate health services should be provided. Health personnel should therefore be available for ongoing surveillance (i.e., monitoring a life-long follow-up of the patient's medical, physical, and psychosocial health).

## Figures and Tables

**Table 1:** Demographic data.

	Number
Gender	
Female	3
Male	7
Age	
18–25	3
26–35	3
36–50	2
51–60	2
Diagnosis	
Osteosarcoma	2
Ewing's sarcoma	5
Chondrosarcoma	3
Time of diagnosis	
3–5 years ago	5
6–10 years ago	5
Treatment	
Surgery	10
Chemotherapy	7
Radiation	2
Amputation	1

**Table 2:** Key findings extracted from the individual stories.

	Participant									
	P1	P2	P3	P4	P5	P6	P7	P8	P9	P10
Age-group	>35	<35	<35	<35	>35	>35	<35	<35	>35	<35
Gender	M	M	M	M	F	F	F	M	M	M
Time since diagnosis (years)	5	7	8	4	6	3	4	8	5	7
Consequences										
Limping	x	x	x	x	x	x	x	x	x	x
Daily use of crutch/stick			x		x	x	x		x	x
Daily use of wheelchair									x	x
Problems with balance		x	x	x	x	x	x	x	x	x
Colostomy										x
Urostomy										x
Pain affecting daily life		x		x	x	x			x	x
Fatigue influencing daily life		x		x	x	x	x		x	x
Daily practical challenges		x	x	x	x	x	x		x	x
Impaired physical activity	x	x	x	x	x	x	x	x	x	x
Lost important hobby	x	x		x			x		x	
On disability benefit					x	x				x
Experienced positive growth		x	x	x			x	x		x
